# Protocol for the synthesis of perovskite nanocrystal thin films via *in situ* crystallization method

**DOI:** 10.1016/j.xpro.2023.102507

**Published:** 2023-10-04

**Authors:** Jaume Noguera-Gómez, Pablo P. Boix, Rafael Abargues

**Affiliations:** 1 Instituto de Ciencia de los Materiales, Universitat de València, P.O. Box 22085, 46071 Valencia, Spain

**Keywords:** Physics, Chemistry, Material Sciences

## Abstract

The field of halide perovskites currently faces the challenge of finding an efficient approach for producing highly efficient and stable perovskite nanocrystals (PNCs). Here, we present a protocol for the annealing-free and antisolvent-free synthesis of PNCs. We describe the steps for preparing the PNCs precursor solutions. We then detail the procedures to control crucial processing parameters, such as the role of precursor concentration and the creation of humidity-controlled chambers, which allow achieving precise control over the final nanocrystals size.

For complete details on the use and execution of this protocol, please refer to Noguera-Gómez et al.^1^

## Highlights

• Crystallization of PNCs nanocomposites with an annealing-free and antisolvent-free method
• An *in situ* fabrication method control over the size of the PNC nanocomposites
• *In situ* generation of PNCs nanocomposite thin films without intermediate steps

## Abstract (editor highlights)

Publisher’s note: Undertaking any experimental protocol requires adherence to local institutional guidelines for laboratory safety and ethics.

## Graphical abstract

**Figure:**
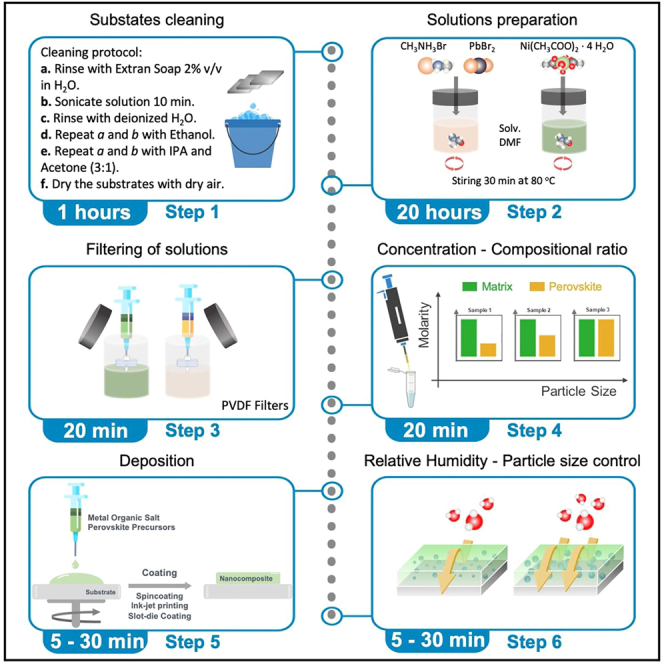

## Before you begin

**Figure 1:**
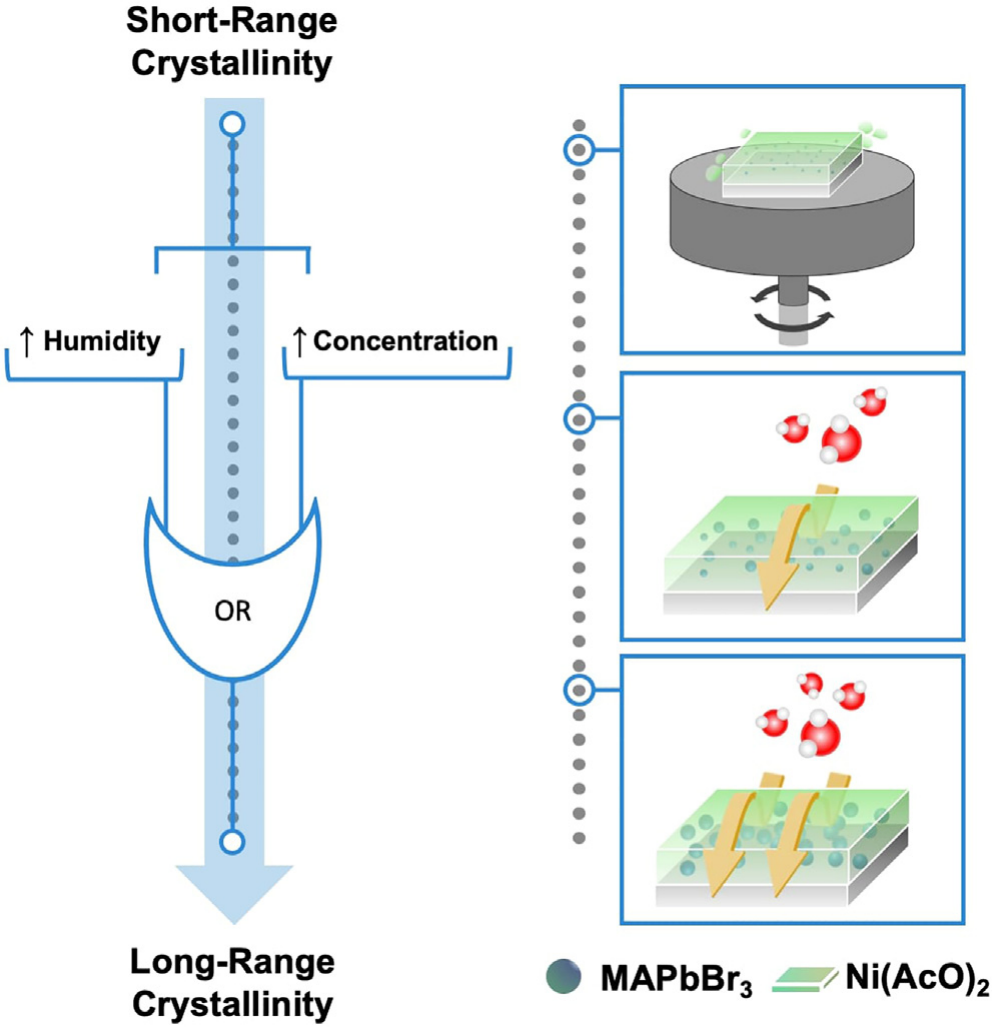
Schematic representation of the nanocomposite fabrication process: an insight into the impact of the concentration and the RH on the PNCs crystallization

This protocol outlines the procedure for the *in situ* synthesis of CH_3_NH_3_PbBr_3_ (MAPbBr_3_) PNCs inside a Ni(CH_3_COO)_2_ (Ni(AcO)_2_) matrix to form a nanocomposite thin film. The final size of the nanocrystals, which ultimately rules the emission properties using quantum confinement, can be precisely controlled by both the relative humidity (RH) during the crystallization process (as discussed in the Spin-coating Deposition subsection) and the precursor concentrations (Figure 1). The synthetic approach serves as a protective measure against the degradation of PNCs by several external factors such as oxygen, UV light, temperature, and moisture.^2^^,^^3^^,^^4^^,^^5^^,^^6^^,^^7^^,^^8^^,^^9^ Additionally, it also allows the synthesis of PNCs of different perovskite compositions, including chloride, bromide, and iodide perovskites and their combinations.

The whole described protocol must be carried out under the appropriate safety conditions regarding each chemical and its derived subproducts (e.g., laboratory facilities, individual protection, and suitable waste treatment). Specifically, lead is a toxic metal that can have detrimental effects on human health. Exposure to lead, whether through inhalation, ingestion, or skin contact, can lead to various health issues. The central nervous system, kidneys, blood, and reproductive system are particularly vulnerable to lead poisoning.^10^ To ensure safety when working with lead-based materials and solvents such as N,N-dimethylformamide (DMF), while following this protocol, it is crucial to follow several precautions. First and foremost, one should always wear personal protective equipment (PPE), such as gloves, goggles, and a respirator, to minimize direct contact and inhalation of lead particles. Work areas should be well-ventilated to prevent the accumulation of lead dust. To ensure safe handling, use, storage, and disposal of the chemical/product as hazardous waste, it is essential to develop a Standard Operating Procedure (SOP) and/or conduct a Job Hazard Analysis (JHA). This procedure should include comprehensive guidelines for handling, storage, and disposal procedures, along with the PPE. Additionally, it is crucial to train workers on reagents hazards, provide them with Safety Data Sheets (SDSs), familiarize them with SOPs and JHAs, and ensure proper labeling of chemicals and lead products to promote clear identification and minimize potential risks. By adhering to these safety instructions, individuals can significantly reduce the potential health hazards associated with lead exposure.

## Key resources table

**Table:** 

REAGENT or RESOURCE	SOURCE	IDENTIFIER
**Chemicals, peptides, and recombinant proteins**		

Methylammonium bromide (MABr), >99.5%	TCI	CAS 6876-37-5
Lead bromide (PbBr_2_), >98.0%	Sigma-Aldrich	CAS 10031-22-8
*N,N*-dimethylformamide (DMF), anhydrous, 99.8%	Sigma-Aldrich	CAS 68-12-2
Nickel (II) acetate tetrahydrate (Ni(AcO)_2_), 98%	Sigma-Aldrich	CAS 6018-89-9
Hydrochloric acid (HCl) 35%, technical	VWR Chemicals	CAS 7647-01-0
2-Propanol (IPA) ≥99.5%	Sigma-Aldrich	CAS 67-63-0
Ethanol ≥99.5%	Sigma-Aldrich	CAS 64-17-5
Acetone, ACS reagent ≥99.5%	Sigma-Aldrich	CAS 67-64-1
Hellmanex III	Sigma-Aldrich	N/A
Inorganic Salts (Table 1)	Sigma-Aldrich or similar	N/A
0.22 μm pore size, 33 mm diameter, Millex-GV Durapore® (PVDF) membrane, hydrophilic	Sigma-Aldrich	N/A
Micropipette 20–200 μL	Thermo Fisher Scientific	11875762
Micropipette Tips 20–200 μL	Thermo Fisher Scientific	11923446
Humidity chambers Multiroir Controlec TOPBOX Plastic Storage Boxes	Thermo Fisher Scientific or similar	15889465
Carbon coated TEM grid	Sigma-Aldrich	N/A
Glass substrate – Microscope slides (76 × 26 mm)	VWR Chemicals	N/A

**Other**		

Ossila Ozone Cleaner	Ossila	https://www.ossila.com/en-eu/products/uv-ozone-cleaner
Spin-coater	Laurell WS-650 Model	http://www.laurell.com/spin-coater/?model = WS-650-23B
Dry-Glovebox, SICCO	SICCO	https://es.vwr.com/store/product/12566364/glove-box-sicco
Thermo-hygrometer Fisherbrand™ TraceableGO™ Bluetooth Datalogging Hygrometer	Thermo Fisher Scientific	15-079-679
Heating / Shaking Dry Bath	Thermo Fisher Scientific	https://www.thermofisher.com/es/es/home/life-science/lab-equipment/dry-baths/heating-shaking-dry-baths.html
Analytical balance	Waagenet	https://eu.waagenet.de/gram-fv/?gclid = CjwKCAiArY2fBhB9EiwAWqHK6jo9lAP9oX-MllvTPeHNtBKoVO5kwUi6_Duudm2eqrA-JWJjTXvHrBoCiesQAvD_BwE
Spectrophotometer Flame-S-VIS-NIR *for PL*	Ocean Insight	https://www.oceaninsight.com/blog/flame-series-general-purpose-spectrometers/
Spectrophotometer UV-2501PC, UV-Vis *for Abs*	Shimadzu	N/A
Blue Laser 404 nm CW GaN	Optoelectronics Tech. Co., Ltd	N/A
D8 Advanced X-ray diffractometer (XRD)	Bruker	N/A
JEOL JEM 1010 Transmission electron microscope (TEM)	JEPL, Tokyo, Japan	N/A
3D Printer – DIGILAB 3d45	Dremel	N/A
Nitrogen Glovebox	MBraun	N/A
Transmission electron microscopy (TEM)	JEOL JEM 1010	N/A

***Note:*** All the chemical reagents can be obtained from different providers as long as they keep the purity degree.

**Table 1:** Inorganic salts for the generation of different RH atmospheres within the Humidity Chambers

Saturated salt solutions	Temperature °C							
	5	10	15	20	25	30	35	40
	Relative humidity, % over the salt solution (*%*)							
Lithium chloride - LiCl	16	14	13	12	11	11	11	11
Potassium acetate - CH_3_CO_2_K	25	24	24	23	23	23	23	23
Magnesium chloride - MgCl_2_	33	33	33	33	33	32	32	32
Potassium carbonate K_2_CO_3_	47	47	45	44	43	42	41	40
Magnesium nitrate - Mg(NO_3_)_2_	54	53	53	52	52	52	51	51
Sodium Chloride - NaCl	76	76	76	75	75	75	75	-
Cupric chloride - CuCl_2_	65	68	68	68	67	67	67	67
Ammonium nitrate - NH_4_NO_3_	-	75	70	67	64	60	53	-
Ammonium sulfate - (NH_4_)_2_SO_4_	82	82	82	81	81	81	80	80
Potassium chloride - KCl	88	87	86	85	84	84	83	82
Potassium sulfate - K_2_SO_4_	98	98	98	98	97	97	97	96

## Materials and equipment

Given example of stock solutions for the PNCs nanocomposite thin films.

**Table:** 

Reagent	Final concentration	Amount
MAPbBr_3_	From 1 to 0.1 M	1 mL
Ni(AcO)_2_	2 M	2 mL
DMF	N/A	1 mL
**Total**	**0.25:1** MAPbBr_3_:Ni(AcO)_2_ (M)	**4 mL**

***Note:*** It is preferable to prepare the final stock solution of the nanocomposite once for each experiment to avoid premature aging caused by the precursors' interaction in the solution.

## Step-by-step method details

### Solution and material preparation (precursor solution)

**Timing: 1 h**

This section outlines the preparation of the two primary precursor solutions. These solutions are meticulously mixed to achieve a transparent solution, which serves as the desired outcome and is then ready for the subsequent deposition step.

We consider MAPbBr_3_:Ni(AcO)_2_ with a 0.25:1 M ratio for the reference case scenario. For the preparation of that ratio.

1. Preparation of the precursor matrix (Ni(AcO)_2_) solution.
  a. Prepare 2 mL of Ni(AcO)_2_ 2 M by adding 2 mL of DMF into a vial with 4 mmol of Ni(AcO)_2_ (0.995 g). Store at room temperature approx. 20°C and ∼40% RH.
    i. Heat the mixture to 70°C and stir continuously in a dry-block bath for 10 min.
    ii. Once finished, filter the solution using a 5 mL syringe with a 0.2 μm PVDF filter.
2. Preparation of MAPbBr_3_ perovskite precursor solution.
  a. Prepare a 2 mL 0.5 M MAPbBr_3_ solution:
    i. In one vial, dissolve MABr 1 mmol (0.112 g) in 2 mL of DMF by stirring for 20 min.
    ii. From the previous vial, take 1 mL of the solution and add it to a second vial containing 1 mmol (0.367 g) of PbBr_2_.
    iii. Filter the solution from step (ii) using a 5 mL syringe with a 0.2 μm PVDF filter. Store the filtered solution at room temperature approx. 20°C and 40% (RH).
3. Preparation of MAPbBr_3_:Ni(AcO)_2_ solution.
  a. Mix 1 mL of step *two* solution with 1 mL of step *one.*
  b. Stir the vial containing the mixture for 10 min.
  c. Store the filtered solution at room temperature approx. 20°C and 40% (RH).

***Note:*** Experiments exploring different ratios to obtain varying particle sizes were conducted, primarily focusing on various ratios MAPbBr_3_ by diluting with DMF. In this experiment, the Ni(AcO)_2_ matrix concentration was maintained at 1 M (see Figure 1).

**CRITICAL:** The matrix and perovskite solutions exhibit long-term stability, maintaining their integrity and properties for up to 3 months when stored at normal laboratory conditions (approx. 20°C–50% RH).

### Substrates cleaning

**Timing: 1 h**

In this section, we detail the steps involved in ensuring the cleanliness of the substrates prior to their use in the deposition step. Thorough cleaning procedures, including solvent cleaning, rinsing, and drying, are implemented to eliminate any unwanted particles or residues. By effectively cleaning the substrates, we aim to provide a pristine surface for optimal adhesion and wettability of the surface.

4. Clean the glass slide substrates.
  a. Sonicate the substrates (approx. 25 × 25 mm) in a 3 M HCl solution (35 vol%, VRW Chemicals) for 10 min.
  b. Clean with a toothbrush and 2% Hellmanex solution in water.
  c. Immerse in an ultrasonic bath containing a 2% Hellmanex (supplier) solution in water for 30 min.
  d. Rinse thoroughly with deionized water.
  e. Rinse with ethanol (supplier).
  f. Immerse in an ultrasonic bath containing IPA and Acetone (supplier) in a 3:1 ratio for 15 min.
  g. Dry the substrates using moisture and dust-free compressed air.

### Spin-coating deposition

**Timing: 5–30 min**

The deposition with a spin coater enables the efficient removal of the excess of solution and drying of solvents, while also facilitating the formation of a thin film with the desired properties adjusting the spinning parameters.

**Figure 2:**
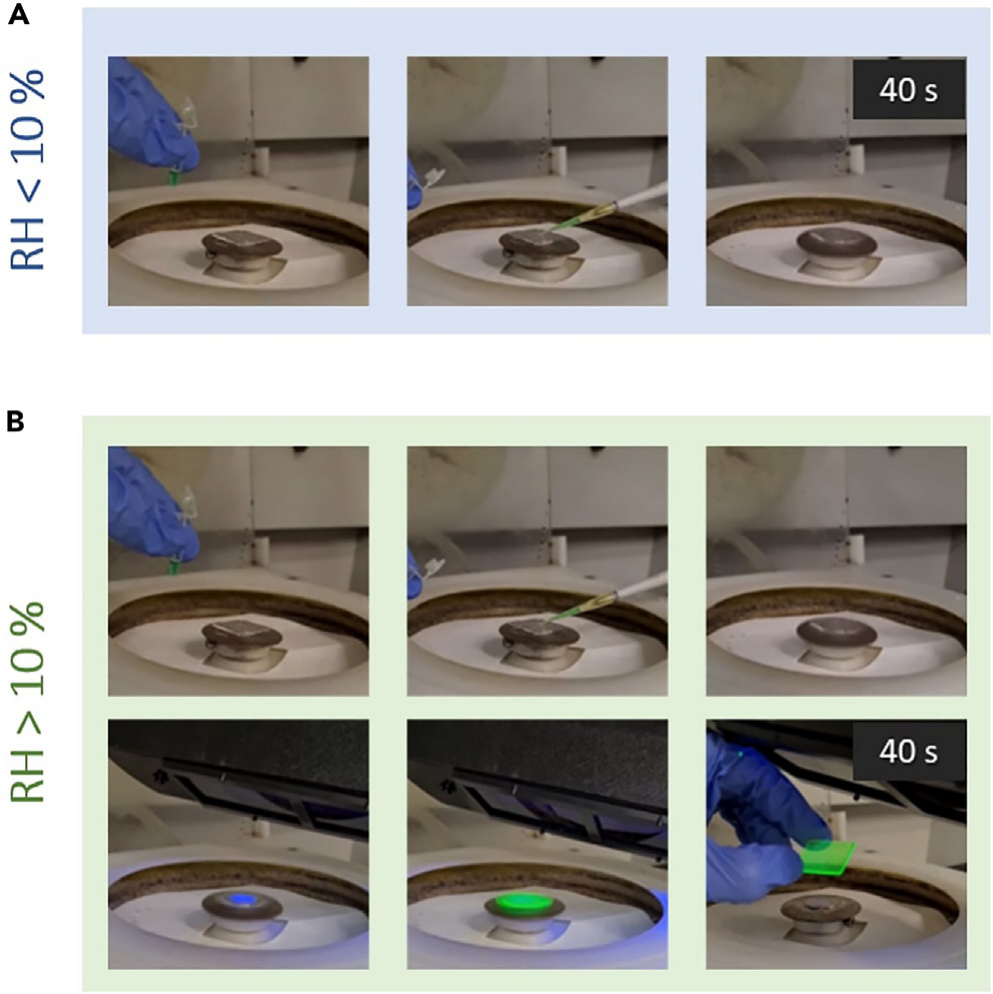
PNCs nanocomposite deposition-frames in a controlled ambient RH (A) RH < 10%. (B) RH > 10%.

**CRITICAL:** As the process strongly depends on humidity, the deposition can be carried out either inside a dry-glovebox (SICCO) supplied with moisture-free air or under ambient laboratory conditions (approx. 40–50% RH and 20°C). The final size of the synthesized PNCs (Figure 2) will be determined by the RH chosen for the deposition process.

***Note:*** With this procedure, the resulting film thickness is in the range of 300 nm. Although, the spin-rate primarily controls the film thickness that influences the absorption properties, while the final size of the PNCs is less influenced by the spin-rate and more dependent on other factors.

### Depositing within a dry box

**CRITICAL:** All the necessary instruments must be placed within this dry box for at least 20 min before the deposition and under moisture-free air.

The single-step synthesis process only consists of depositing the nanocomposite precursor solution onto a glass.

5. Operate with the spin-coater as described:
  a. Center the glass substrate (treated with ozone in the spin-coater and set the operating parameters to 3000 rpm for 40 s with an acceleration ramp of 2000 rpm/s.
  b. Drop 70 μL of the solution in the center of the glass substrate in static using a Micropipette and start the spin-coater program.

Once the program is finished, place the sample within a previous-fabricated humidity chamber (as described below in the humidity chambers building subsection) and for the appropriate time to trigger the crystallization with specific PNC size (see Noguera-Gómez et al. 2022).

### Depositing in standard laboratory conditions

**CRITICAL:** Depositing outside of a dry box will propitiate the crystallization of the PNCs when exposed to humidity conditions with a RH difference > 10%) (see Figure 6).

***Note:*** In this scenario, please perform the steps outlined in the previous instructions but conduct the procedure outside the dry box. The relative humidity and temperature (laboratory conditions) during the deposition step will influence the final size of the PNCs dramatically.

### Humidity Chambers Building

**Timing: 1 h**

In this section we provide an overview of the design and construction of the humidity chambers used to generate precise humidity levels ranging from 10 to 100% RH.^11^

Humidity chambers have been designed to generate a specific humidity level from 10 to 100% RH by using a supersaturated solution of different inorganic salts. The salt solutions are used because they have a well-defined equilibrium vapor pressure at a given temperature, which makes it possible to generate a specific level of relative humidity inside a chamber.

**Figure 3:**
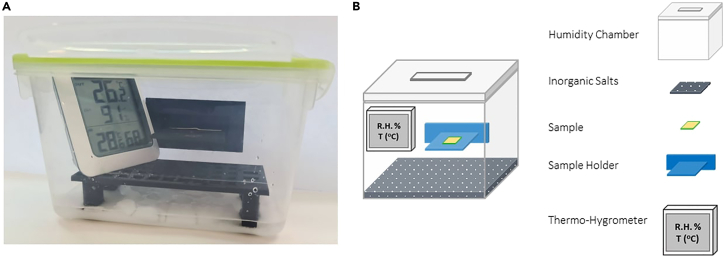
Humidity Chamber (A) Real-built Humidity Chamber. (B) Schematic representation of a Humidity Chamber.

The humidity chambers consist of 4 essential parts (Figure 3):

Polypropylene Box with an airtight lid.

Saturated Salt Solutions.

Sample Tray Holder (Data S1- STL Printable File).

Thermo-Hygrometer.

**CRITICAL:** To guarantee the desired relative humidity level, once the supersaturated salt solution is placed in the chamber, the chamber is closed with an airtight lid. The RH is monitored with a hygrometer. The equilibrium vapor pressure is reached after 30 min for a 20 × 20 × 20 cm box. Plastic trays were designed and attached to one of the side walls of the chamber to enable the insertion of samples without breaking the equilibrium. The trays (Data S1- STL Printable File - Sample Tray Holder for Humidity Chambers) were 3D printed using polylactic acid (PLA) with a 100% infill.

***Note:*** Adding multiple trays does not negatively impact the establishment of equilibrium within the vessel.

The relative humidity is determined by the partial vapor pressure of the salt solution mixture at a given temperature once the equilibrium is reached. To prepare a humidity chamber, we use different inorganic salts available according to the desired RH (see Table 1).

6. Preparation of saturated salt solutions involves the following steps:
  a. Select the appropriate salts based on the RH range being investigated (Table 1).
  b. Fill the bottom of the chamber with 1 cm layer using the salt from step 1.
  c. To achieve this, slowly add small portions of distilled water while gently agitating to promote a homogeneous distribution with the salt. The final goal is to establish a slushy mixture that ensures a supersaturated state of the salt in aqueous solution.
  d. Upon reaching the supersaturated state, close both the airtight lid and the box trays and wait approximately 30 min to reach the equilibrium vapor pressure (check the RH with a thermo-hygrometer).

**CRITICAL:** The minimum recommended box size is 20 × 20 × 20 cm.

***Note:***Table 1 compiles common inorganic salts as potential candidates for preparing controlled humidity chambers. As previously mentioned, in a state of supersaturation, the containers establish a vapor equilibrium that enables the attainment of relative humidity at the tabulated temperature.

## Expected outcomes

We have developed a method that enables the synthesis of PNC nanocomposites through a novel *in situ* approach, which allows controlling the final particle size. Compared with similar methods, our approach is a facile antisolvent-free and glovebox-free (cost-effective) strategy with excellent emission properties.

**Figure 4:**
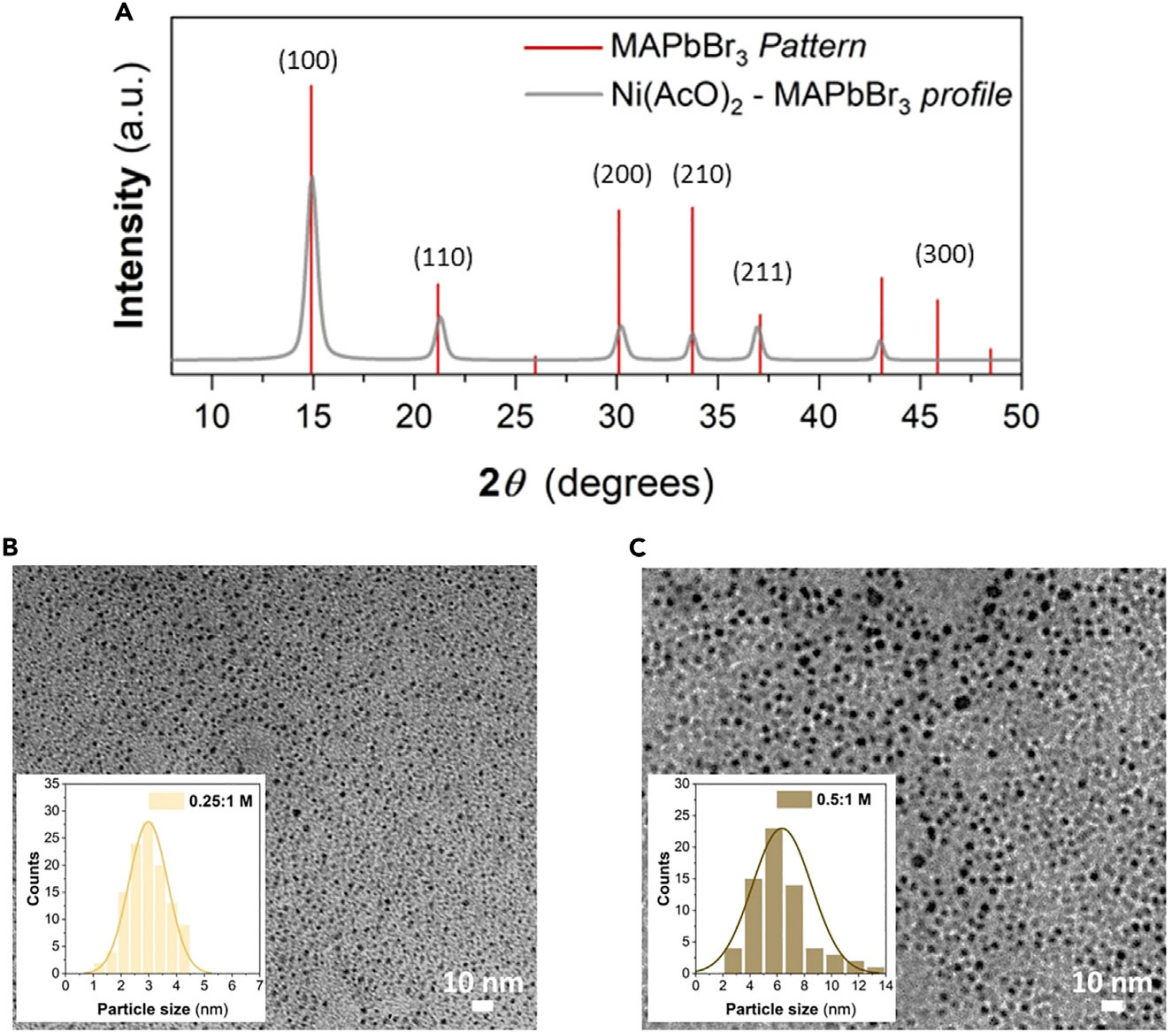
Structural and morphological characterization of nanocomposites (A) XRD diffraction pattern of as-synthesized MAPbBr3:Ni(AcO)2 nanocomposite under room humidity conditions (50% RH). (B and C) TEM images and particle-size distributions for PNC nanocomposites at two different MAPbBr3:Ni(AcO)2 ratios, 0.25:1 and 0.5:1 M, respectively. Reprinted with permission from Noguera-Gómez et al., (2022).

To effectively characterize thin films, both X-ray diffraction (XRD) and transmission electron microscopy (TEM) can be studied to accurately analyze the formation of the PNCs nanocomposites. These techniques allow for assessing the existence of PNCs in the matrices following the prosperous production of PNCs nanocomposites. This evaluation is depicted in Figure 4.

***Note:*** The *in situ* synthesis of MAPbBr_3_ in Ni(AcO)_2_ can be confirmed by the X-ray diffraction (XRD) diffractogram (Figure 4A), which reveals diffraction peaks corresponding to the cubic Pm3m crystal phase of MAPbBr_3_ (JCPDS no. 00–0105) in the nanocomposite. Notably, films prepared under RH <10% exhibit no crystalline patterns, indicating that MAPbBr_3_ does not form at low RH. This finding is consistent with the negligible optical properties observed in the absorbance and PL spectra (Figure 6C). However, upon exposure of the films deposited at RH <10% to the ambient atmosphere (RH >15%), the crystallization process initiates, resulting in a gradual color change and an increase in the excitonic absorption band and PL.

For the film crystallization processes under the same RH, higher perovskite precursors concentration leads to larger PNC size, as confirmed by transmission electron microscopy (TEM) measurements (Figures 4B and 4C). At a lower concentration of MAPbBr_3_ (0.25 M), smaller PNC sizes are observed, with a distribution centered around 2–3 nm, while larger amounts of MAPbBr_3_ (0.5 M) result in larger PNCs, with a distribution centered around 6–7 nm.

**Figure 5:**
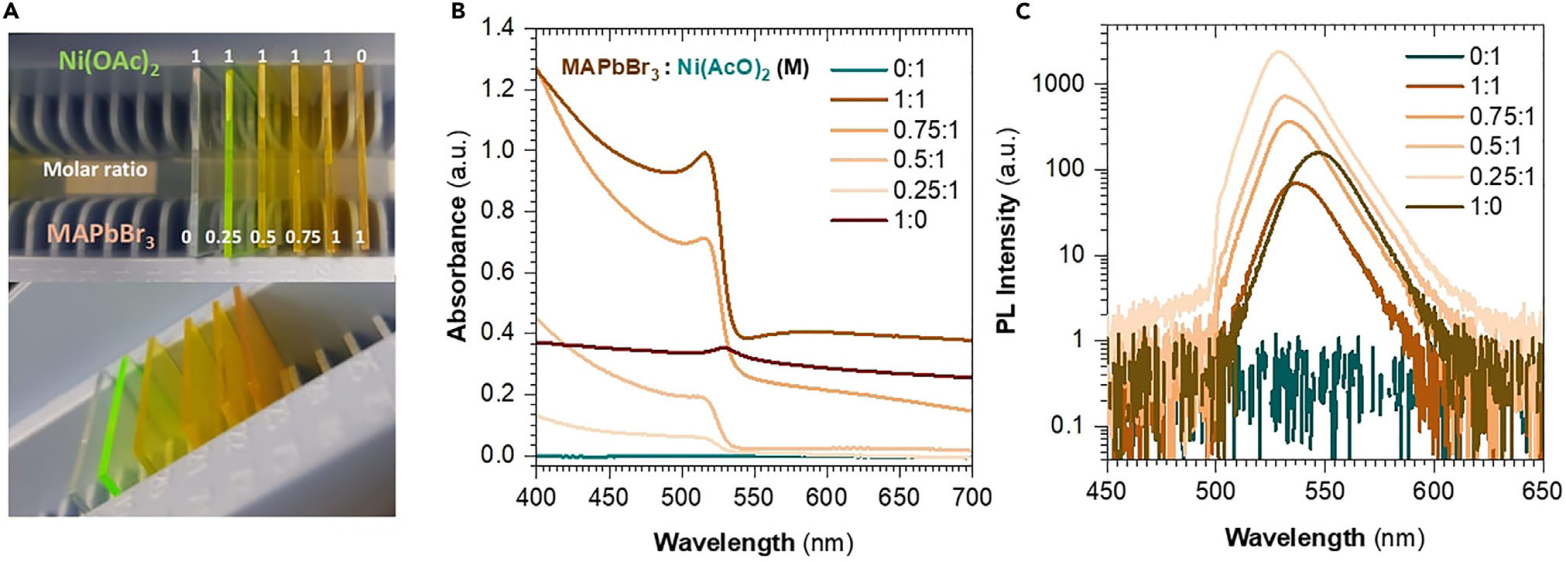
PNCs nanocomposite thin films (A) Photograph of the PNCs nanocomposite thin films at different concentration ratios of MAPbBr3 (Ni(AcO)2 was fixed to 1 M). (B and C) Absorption and PL Spectra of the thin films, respectively.

The role of concentration in the formation of the PNCs nanocomposite is crucial. Therefore, when the concentration ratio of the PNCs precursor is varied with respect to the matrix, the expected outcome can be studied as depicted in Figure 5. After a successful synthesis, the optical properties of the resulting thin films fabricated at 50% RH are characterized (absorption and PL spectra). The equipment needed for these characterizations can be found in the key resources table.

Note: As greater amounts of MAPbBr_3_ are loaded within the matrix (Figure 5A), up to the bulk phase (1:0), the absorption spectra (Figure 5B) show a clear trend of increasing absorption peaks that shift towards lower energies. In addition, the photoluminescence (PL) response (Figure 5C) depicts a redshift, transitioning from wavelengths ranging from 515 nm in the sample with a lower amount of MAPbBr_3_ (0.25:1 M) to 550 nm in the pure bulk MAPbBr_3_ sample. This shift is attributed to the variation in average particle sizes within the matrix (as the TEM Images supported in the previous subsection) with funneling effects.^1^^,^^12^

**Figure 6:**
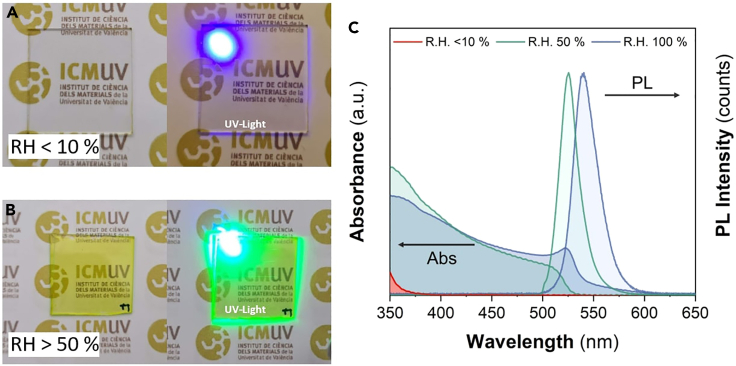
Humidity Influences (A and B) Photographs of nanocomposite thin films under different RH conditions, <10% and >50%, respectively. (C) PL and absorption spectra of the nanocomposite film fabricated with a MAPbBr3:Ni(AcO)2 ratio of 0.25:1 M and crystallized under varying RH conditions.

Alternatively, to the precursors’ concentration effect, RH also impacts the crystallization dynamics. The PL and absorption spectra of perovskite nanocomposite films with a MAPbBr_3_:Ni(AcO)_2_ ratio of 0.25:1 M are shown in Figure 6. These spectra are measured after exposing the films to various RH levels for 60 min. The absorption edge and emission peak exhibit a redshift as RH increases from 50% to 100%. The most prominent distinction between the two absorption spectra is the clear excitonic absorption peak at 525 nm when the film is exposed to a RH of 100%, unlike the one exposed to 50%. Furthermore, when exposed to higher RH, a PL band shift is observed from 525 nm (RH of 50%) to 540 nm (RH of 100%). These results are in perfect accordance with the TEM Images discussed above.

**CRITICAL:** It is noteworthy that while ambient humidity affects the crystallization process, the optical properties of the nanocomposite film are stable once a crystallization plateau is reached. The stable conditions can be reached after different periods of exposure to certain RH conditions for each precursor concentration.

**Figure 7:**
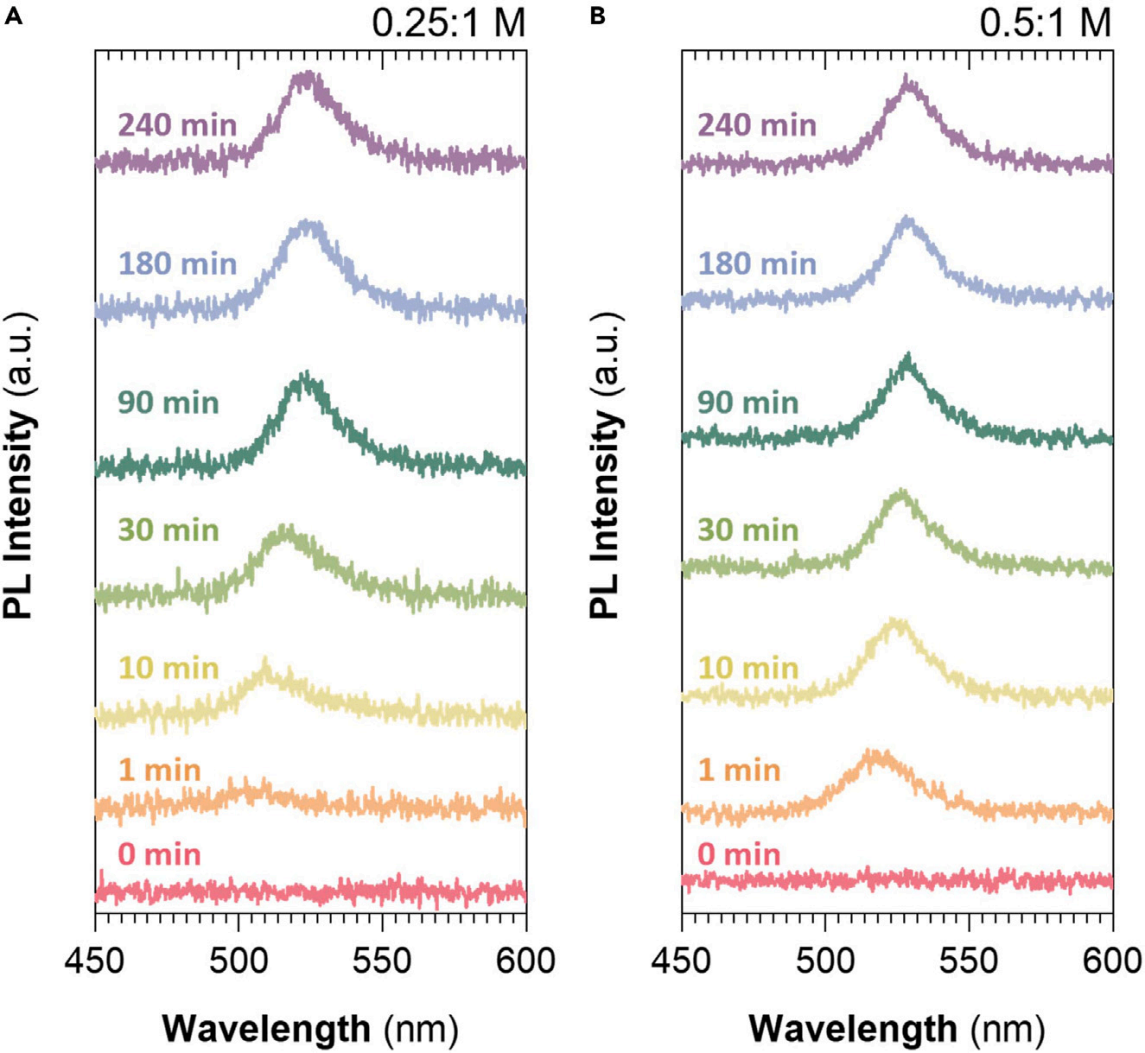
Crystallization dynamics are monitored by the evolution of the sample photoluminescence to illustrate the concentration and humidity influence on crystallization (A and B) 0.25:1 and 0.5:1 M ratio at 50% RH over time, respectively.

Thus, it becomes essential to understand the crystallization dynamics to tune the optoelectronic properties of the perovskite thin film nanocomposites in a stable way. While the exposure to higher ambient humidity results in faster perovskite crystallization, the precursor concentration can also affect this reaction. The impact of an RH of 50% is investigated on two different samples with different precursors concentration immediately after preparation within a dry-ambient glovebox. The *in situ* synthesis of PNCs within the Ni(AcO)_2_ is monitored by their optical properties at different times, as shown in Figure 7. Our observations indicate that the reaction is more abrupt when the perovskite concentration is higher (0.5 M), working with the matrix fixed at a concentration of 1 M. This phenomenon may occur due to the existence of more nucleation centers in the medium, which is derived from the higher perovskite concentration. Besides, a plateau of emission is observed at shorter times for higher concentrations (0.5 M), which is related to an almost complete reaction of the perovskite precursors within the Ni(AcO)_2_ matrix.

## Limitations

One of the limitations is that precursor solutions, especially with sol-gel precursors such as the Ni(AcO)_2_, may eventually precipitate after several months. Hence, the filtration step plays a critical role in ensuring the maximal longevity of the precursor by removing unsolved particles that can act as nucleation centers.

## Troubleshooting

The approach of synthesizing PNC nanocomposites *in situ* can potentially establish a foundation for producing optoelectronic devices on a large scale with improved properties. It can also serve as a framework for tuning the bandgap through the crystallization dynamics control of the PNC size. To ensure successful synthesis of the PNC nanocomposites using the *in situ* approach, it is important to be aware of potential issues that may arise during the process. The following troubleshooting section aims to address common challenges and provide possible solutions.

### Problem 1 – Particle Agglomeration

Aggregation, sudden aging, or clustering of the sol-gel matrix solution, leading to poor dispersion and heterogeneous uniformity (solution and material preparation (precursor solution) Subsection).

### Potential solution

If any of the solutions (MAPbBr_3_ or Ni(AcO)_2_) unexpectedly precipitates, it is advisable to replace it with a fresh one, even if it was expected to have a longer shelf life of three months. This ensures the reliability and consistency of the solution’s properties for the next steps.

### Problem 2 – Solutions preparation

Undissolved reagents at the bottom of the vial after following the instructions for the preparation of Ni(AcO)_2_ (solution and material preparation (precursor solution) Subsection).

### Potential solution

To ensure optimal homogeneity and complete solubilization of the solution, gently heat the mixture while stirring in the dry-block bath. This will promote better mixing and aid in the dissolution process. If needed increase the heating temperature of the dry-block bath up to 80°C and heat for 5 additional min. Once done, proceed with filtration to remove any remaining particulates or undissolved components.

### Problem 3 – Poor wettability of substrates

Despite meticulously adhering to the cleaning protocol, the substrates' surface wettability remains inadequate, leading to aggregation or inadequate distribution of the solution on the surface (substrates cleaning Subsection).

### Potential solution

Perform an ozone or a plasma treatment (Ossila Ozone Cleaner or a similar alternative) for 10 min before the nanocomposite deposition. Alternatively, deposit through spin coating 1 mL of DMF and then deposit the nanocomposite precursor solution.

**CRITICAL:** Attention should be given to the fact that ozone is a hazardous gas. It is recommended to perform ozone treatment within a fume hood or a similar controlled environment.

### Problem 4 – Humidity chambers equilibrium

If the humidity chamber fails to reach equilibrium within the desired time frame, resulting in an incomplete stabilization of the humidity level (humidity chambers building Subsection).

### Potential solution

Extend the duration by leaving the humidity chamber for an additional 10–20 min. This allows to reach vapor pressure equilibrium with the desired humidity value and ensures that the system achieves a stable and reproducible experiment environment before proceeding. Additionally, in case of frequent chamber openings that disturb the equilibrium, promptly close the gate, and patiently wait until the desired RH is restored.

### Problem 5 – TEM samples

TEM samples are prepared by dispersing a smashed thin film on top a Carbon coated TEM grid. When using a high concentration of dispersed nanocomposite film, it can lead to difficulties in visualizing individual PNCs due to the formation of agglomerates (expected outcomes Section).

### Potential solution

To ensure proper visualization of the PNCs, it is recommended to work with diluted solutions for depositing onto the TEM grid.

## Resource availability

### Lead contact

Further information and requests for resources and reagents should be directed to and will be fulfilled by the lead contact Pablo P. Boix (pablo.p.boix@uv.es).

### Materials availability

All reagents produced in this study are accessible through the lead contact.

### Data and code availability

All data is available upon contacting the authors.

## Supplemental information

Data S1. STL Printable File - Sample Tray Holder for Humidity Chambers, related to Figure 3 in the Humidity Chambers Building Section
